# Does Coercive Process Play a Role in Teen Dating Violence?

**DOI:** 10.3390/adolescents6010023

**Published:** 2026-02-18

**Authors:** Danielle M. Mitnick, Amy M. S. Slep, Stacey Tiberio, Kelly A Daly, Richard E. Heyman, Michael F. Lorber

**Affiliations:** 1 New York University; 2 Oregon Social Learning Center

**Keywords:** teen dating violence, coercion, coercive process

## Abstract

Coercion theory posits that a vicious cycle of negative reinforcement traps both parents and children, shaping young children to become stably aggressive in conflicts with their parents. Research on intimate partner violence has found some evidence supporting the application of coercion theory in explaining aggressive escalation in adult conflicts as well. It is unclear whether the same processes are at play during teens’ early dating relationships. On the one hand, dating aggression emerges as soon as dating relationships do. On the other hand, we also know that if aggression presents extremely early in relationships, dissolution is the most likely path. We explored the role of coercion in 209 teen dating couples who were observed in a laboratory series of problem-solving discussions and analogue conflict tasks. All data were coded by trained coders blind to hypotheses. Analyses suggest that negative reinforcement of hostility is indeed significantly associated with both psychological and physical aggression in an adolescent dating sample. Implications for prevention and intervention are discussed, as well as developmental processes that may contribute to dating aggression in light of our findings.

## 1. Introduction

Teen dating violence (TDV) affects one in five adolescents[1], and understanding this phenomenon requires attention to dyadic processes, yet this aspect remains relatively overlooked. Research indicates that patterns of interaction between partners may contribute to the development of TDV. Notably, most TDV is mutual [2]. There is evidence of assortative pairing in which aggressive boys and girls seek one another out [3–7]. Furthermore, partners shape each other’s behavior [8,9]; one partner engaging in TDV increases the risk of the other responding in kind [3,10]. TDV tends to persist more strongly in young couples who stay together compared with those who form new partnerships [11–13], suggesting that some of the variability in TDV stems from relationship dynamics, not solely from individual partner traits.

A focus on interpersonal processes in TDV is particularly warranted because adolescents’ desire for romantic connection tends to develop ahead of the relational skills needed to manage these new experiences effectively [14]. Unlike adults with relationship histories, adolescents are navigating romantic conflicts for the first times, making developemtnal context essential to understanding their conflict processes. Rates of dating increase steadily across teenage years, with roughly half of 15-year-olds reporting romantic involvement, climbing to more than 70% by age 18 [15]. Adolescents in relationships are especially vulnerable to negative mood states [16], and disagreements with partners occur frequently and are experienced as highly stressful [17,18]. At the same time, capacities for self-regulation are still developing [19,20], which contributes to the heightened emotional intensity of teenage relationships [14]. Managing conflict in these contexts introduces adolescents to challenges such as jealousy, heightened emotion, and intimacy concerns, and the skills to address them often evolve only through repeated relational experiences [21,22].

Thus, scholars have increasingly emphasized the need to examine dyadic processes that contribute to TDV [23–26],yet the literature on the relational foundations of adolescent dating aggression is limited compared with research on individual-level risk factors. Much of the work on TDV has centered on ways in which adolescents’ backgrounds and contexts outside their romantic relationships (e.g., family aggression [27], genetics [28], anger [29], attitudes toward aggression [30], exposure to aggressive peers [31]) shape behavior within them. In addition, the majority of studies focus on individuals rather than couples (e.g., [32,33]). Importantly, few investigations have used observational methods to examine interactions between adolescent partners (e.g., [4]). Observational approaches are critical for capturing relationship processes in real time[34,35], offering insights into subtle interpersonal behavioral dynamics that self-report methods cannot.

### 1.1. Coercive Process as an Explanation for TDV

Despite the limited research, theory has been proposed to explain the likely role of couple relationship dynamics in TDV (e.g., [36,37]). In the present article, we empirically evaluate the relevance of coercion theory to TDV. According to coercive process — first noted in the parent-child interactions of children on deviant, aggressive developmental trajectories [38]—people may inadvertently train one another that aggressive conflict tactics (e.g., insults, hitting) work to their advantage. This process is referred to as “basic training” [39]. When aggressive tactics work better than more agreeable ones, they are disproportionately likely to be repeated in the future. Likewise, giving in to aggressive escalation is rewarded when backing down in the face of hostile behavior successfully ends or reduces conflict. Because sometimes one person “wins” by escalating and other times the other does, coercive conflict can be marked by initial mutual escalation of hostilities. Each partner attempts to out-coerce the other into backing down. The relative probability of aggressive escalation being reinforced compared with the probability of more agreeable, de-escalating behaviors being reinforced determines which behaviors will increase over time and be favored [40]. As Patterson states, each person is both the “victim and architect of a coercive system” [41]. Such interactions may eventually lead to TDV.

It is important to note that while coercion theory has demonstrated relevance across parent-child, sibling, and adult intimate partner relationships, adolescent dating relationships represent a distinct developmental context. Adolescents are navigating romantic conflicts for the first time with limited experience, while simultaneously managing neurobiological changes [19,20], heightened emotional reactivity [42], and still-developing self-regulatory capacities [19,20]. Thus, while coercive processes may operate in adolescent relationships, they may manifest differently than in adult partnerships where individuals have more relational experience and fully developed executive function.

To date, parent-child and sibling coercive process has been shown to relate to child aggression [41,43–46]. Relatedly, peer reinforcement is implicated in other forms of externalizing youth behavior [47–49]. Given these findings and the strong social embedding of adolescent behavior [50], reinforcement patterns are likely relevant to adolescents’ conflicts with their partners.

Coercive conflict has a long history in theories of men’s violence toward women [51–53], though these frameworks were developed primarily for adult relationships. We have previously found support for the relative reinforcement of escalation in adult couples[54], though it remains unclear whether similar processes operate in adolescent relationships given developmental differences.. A smaller group of scientists has explicitly hypothesized the role of some form of coercive process in adolescent couples’ relationships [26,55–58]. Yet only recently has the core Pattersonian “basic training” hypothesis of negative reinforcement for aversive behavior been examined in relation to TDV. In the most relevant study to date, Capaldi and colleagues [4] examined late adolescent couples either in their senior years of high school or within 2 years of graduation (17–20 years). Consistent with the coercion model, Capaldi found greater verbal hostility and reciprocity of low-level physically aggressive behaviors in violent couples [4]. However, this study did not examine reinforcement for hostility specifically.

Although negative reciprocity is an expected product of basic training, it is not direct evidence of the process itself and can be viewed through other conceptual lenses. For example, frameworks developed for adult relationships, such as Sabourin’s [59] emphasis on power and control, stipulate that couples negotiate issues of dominance and submission in an ongoing fashion. From this view, mutual negative behavior is a struggle for dominance — one attempt for control is met with another as each person tries to gain the upper hand. Reciprocal hostile and physically aggressive behaviors could reflect a retaliatory or even an “escalation prevention” motivation [4,60]. Additionally, the link between teen couples’ negative reciprocity and TDV may be due to a failure of self-regulatory processes and/or the immaturity of their conflict management skills, rather than a meaningfully dyadic process. As discussed above, it is developmentally normative for teens to have limited capacities for “hot executive function,” in which they take deliberate action to manage their behavior in emotion-laden encounters [20]. Further, relative to their peers, teens who have lower self-regulatory capacities or poorer conflict management skills may have difficulty inhibiting aversive behavior in favor of more prosocial ones (so-called “editing-out-the-negative” [61]). These developmental considerations suggest that patterns of negative reciprocity in adolescent couples may reflect maturational processes rather than the reinforcement-based mechanisms described in adult coercion models. Given these plausible alternative explanations for the findings suggesting negative reciprocity is linked with TDV, it is important to isolate the role of reinforcement for escalating aversive behavior as specified by coercion theory [40]. Without this, we cannot conclude that coercive process is linked to TDV.

Another study that supports examining the role of coercive process further found that the degrees of perceived conflict and sarcasm in a laboratory couples observation of 14–18-year-olds was associated with boys’ and girls’ TDV [55]. Boys’ violence was also more frequent in couples who reported that more “giving in” had occurred in the interactions. Giving in is an important part of the basic training process. However, this study’s methods did not examine reinforcement for giving in, and thus, does not directly speak to basic training per se. Moreover, the concordance between couples’ perceptions of giving in and giving in as specified by coercion theory is unclear. Accordingly, there are only hints in the existing literature that basic training in aversive behavior via negative reinforcement (i.e., basic training) might explain TDV.

### 1.2. The Present Investigation

We conducted what we believe to be the first test of the basic training hypothesis in TDV, our primary aim and novel contribution. Specifically, we tested the core theoretical assumption that TDV would be positively associated with the relative reinforcement of escalations in hostile vs. nonhostile behaviors via partner’s de-escalations in hostility during conflict (H1). As our secondary aim was to contextualize our findings within the broader literature, we sought to replicate prior findings [4] suggesting positive associations of TDV with the amount of hostile behavior (i.e., the ratio of hostile to non-hostile behavior) displayed during conflict (H2) and reciprocation of hostile behavior (i.e., the tendency for one partner’s hostile behavior to be followed by hostile behavior from the other partner; H3). These replication hypotheses provide important benchmarks for interpreting the unique contribution of negative reinforcement processes beyond general hostility and reciprocity patterns. These hypotheses were evaluated in an observational study of 209 American 14- to 18-year-old high school couples.

Patterson’s original basic training model emphasizes hostile behavior being negatively reinforced by conflict termination or the cessation of aversive behavior from the partner [41]. Ideally, we would operationalize coercive process by examining behaviors reinforced by conflict termination. However, many couples did not experience conflict terminations, and using the original operationalization would involve disregarding much of the data. Therefore, we operationalized negative reinforcement as hostile escalation followed by the partner’s subsequent de-escalation, considering this a micro-temporal proxy that captures the backing down from escalation that characterizes coercive exchanges [54]. This approach tests whether the partner reinforces increases in hostility through immediate yielding behavior, rather than the more ultimate conflict termination. This represents a more microscopic view of coercive process than Patterson’s original formulation but maintains the core theoretical principle of negative reinforcement of escalating behavior.

## 2. Materials and Methods

All recruitment and procedures were approved by the Institutional Review Board at New York University.

### 2.1. Participants

A total of two hundred and nine adolescent couples (*n* = 418 individuals) participated. Screened couples were eligible to participate if: (1) both partners were 14–18 years old; (2) both partners had not yet begun college; (3) they had been dating for ≥3 months; and (4) at least one partner reported recent relationship conflict from a one-item screener.

To recruit teenage participants, research staff stood near public, charter, and private schools, as well as public spaces (i.e., shopping areas, fast food restaurants, and businesses popular amongst adolescents) in New York City and nearby areas in New Jersey (e.g., Jersey City). Recruiters explained the study to interested adolescents and distributed business cards with contact information. Interested adolescents contacted the study team, were screened for eligibility, and parental permission was verbally obtained for adolescents under the age of 18. In addition to face-to-face recruitment, recruiters posted flyers in public spaces with study information, visited schools to provide oral presentations and contact information, and study participants were asked to refer potentially eligible acquaintances via word of mouth, social media, etc. Of the 209 couples recruited, 58.9% (*n* = 123) of couples were recruited from schools, 17.7% (*n* = 37) by referrals from family or friends, 13.4% (*n* = 28) by a recruiter at a public location (i.e., mall, restaurant), 6.7% (*n* = 14) from social media (i.e. Facebook, Instagram), and 3.3% unknown (*n* = 7).

The mean age of study participants was 17.1 years (*SD* = 1.0). Among participants, 48.1% identified as Latino or Hispanic of any race. Non-Latino participants described themselves as African American (19.9%), Asian (10.2%), Caucasian (19.7%), Native Hawaiian or Pacific Islander (0.9%), Mixed Race (2.4%), or Other/Don’t Know (3.8%). Among participants, 52.6% identified as Female, 46.7% as Male, 0.4% as Transgender, and 0.4% as Other. Additionally, 79.2% of participants identified as Heterosexual, 5.7% identified as Homosexual/Lesbian/Gay/Queer, 11.9% identified as Bisexual, and 3.1% were “not sure.” Twenty-three of the couples were same sex (*n* = 18 female-female; *n* = 5 male-male). The mean length of the present relationship was 17.2 months (*SD* = 11.9).

### 2.2. Procedure

Each couple completed a 3-hour laboratory visit in an office building in downtown Manhattan. All observational tasks were video-recorded with 2 Sony HD Color Video Camera EVI-H100V cameras (with an additional third backup camcorder) and stored on our secure network for later coding.

#### 2.2.1. Laboratory visit.

The laboratory visit consisted of a questionnaire battery, approximately 1 hour of observational behavior tasks, and a debriefing. Each participant was paid $35 each (including a $5 surprise bonus).

#### 2.2.2. Questionnaire battery.

Questionnaires were individually administered to each member of the couple in separate rooms, to ensure privacy and prevent collaboration.

#### 2.2.3. Behavioral observation.

The video-recorded behavioral observation protocol involved six tasks designed to elicit conflict behaviors. Tasks were adapted from prior research on adolescent couples [4,62,63], and two novel tasks were developed for this study. Tasks 1 and 6 were always administered first (warm-up) and last (cool-down), respectively.

1. Party planning task (5 minutes; [4]). Participants were asked to plan a hypothetical party together within an unlimited spending limit.
2. General dating issues task (7 minutes; [4]). The couple discussed what they think is important in dating relationships. An accompanying sheet of prompts (e.g., “What are the top 5 most important things in a dating relationship?”) assisted in guiding the conversation.
3. Problem-solving task (≥ 20 minutes). Couples were asked to discuss issues of unresolved conflict in their relationships (e.g., [4,62,63]. Each partner separately completed a 25-item checklist (see Conversation Assessment Tool-Kiddie below) of common issues in adolescent relationships to determine personally relevant problems for discussion. Research staff compiled the three highest-rated issues per partner (ties resolved randomly), onto discussion cards sequenced from highest to lowest importance per partner. Couples engaged in two ten-minute problem-solving discussions — one for each partner’s selected issues — with instruction to discuss the first issue and to move on only to if they were both happy with the resolution. Initiation order was determined randomly and was blocked to ensure even distribution.
4. Lego task (10 minutes). Drawing partly from the group dynamics approach of West and colleagues [64], each couple was asked to construct a complex Lego model using a diagram across the room within an unrealistically brief timeframe. Couples were told to leave the diagram on a distant table, with only one partner permitted to view the diagram at a time. To promote collaboration, each participant was told to keep one arm behind their back. To stimulate competition, a $5 reward was promised to the partner who did the “best job” (although $5 was ultimately provided to both members at the end of the laboratory visit, irrespective of performance). To increase pressure, an audio recording of a ticking clock played, with announcements marking each remaining minute. To further motivate the couple, they were told that most couples were able to complete the design in 6–7 minutes, but they were being given 10 minutes to complete it.
5. Tangram puzzle task (10 minutes). In a novel task, couples were asked to work together to solve a puzzle requiring geometric shapes (e.g., triangles) to be configured to replicate figures shown in a diagram, forming a picture (e.g., a swan). The task consisted of two 5-minute segments, counterbalanced by gender, with each person alternating between the roles of “teacher” and “builder.” The teacher held the diagram and verbally guided the builder on how to construct each figure without pointing at or touching the puzzle pieces. The builder assembled the puzzle based on the teacher’s instruction, without viewing the diagram. To increase pressure, the couple was informed that most couples could complete 2 to 3 puzzles per turn. Furthermore, as with the Lego task, an audio recording of a ticking clock played continuously, with announcements marking each remaining minute. To further motivate the couple, an additional $5 incentive was offered if they could correctly complete two designs in each 5-minute session (though this incentive was provided to all couples at the end of the lab visit, regardless of their performance).
6. Enjoyable memories task (5 minutes; [4]). The couple was asked to discuss good times in their relationship. This task was always last, to facilitate a cool-down if conflict arose during previous tasks.

##### 2.2.3.1 Rationale for observational task design.

The observational protocol was designed to elicit a range of conflict behaviors while maintaining ethical standards for adolescent research. The tasks progress from low-stakes collaboration (party planning) to performance pressure tasks (Lego, Tangram), mirroring demands that couples face in naturalistic settings. The problem-solving discussions are particularly well-suited to capturing conflict processes relevant to TDV, as the adolescents themselves identified the issues as important sources of conflict in their relationships, ensuring ecological validity. The 20-minute problem-solving format has been validated in prior adolescent TDV research[4, 11]). The performance-pressure tasks were designed to create time pressure and communication challenges to mirror realistic adolescent challenges. Although laboratory observations cannot capture all aspects of naturalistic conflict, including physical aggression [4], they offer the advantage of observation of turn-by-turn interaction patterns that participants cannot self-report.

#### 2.2.4. Debriefing.

At the end of the laboratory visit, the couple was debriefed about the deception involved in the Lego and Tangram tasks (i.e., that the tasks are not as easy to solve as we led them to believe and that the “bonuses” would be paid regardless of task performance).

### 2.3. Measures

All measures were completed independently by each member of the couple while they were completing their laboratory visit.

#### 2.3.1. Dating violence.

1. Safe dates physical dating abuse scales (Safe Dates Teen Dating Violence (TDV); [65]). Separate 16-item perpetration (i.e., self-to-partner) and victimization (i.e., partner-to-self) subscales were computed, with items ranging from relatively minor (e.g., slapping) to severe (e.g., choking) forms of physical aggression. The frequency of each behavior in the last year was rated on a 4-point scale from “never” to “10 or more times.” Responses to each item were averaged and item averages were computed for each partner for each subscale. Physical TDV scores were operationalized for each partner as the maximum of her/his reported perpetration and their partner’s reported victimization, thus taking both partners’ reports into account. The Safe Dates TDV measure has exhibited validity and longitudinal stability [66,67]. Safe Dates TDV scores are index scores, not assuming an empirical structure among the items; thus, internal consistency is not reported.
2. Conflict in adolescent dating relationships inventory (CADRI; [68]). The verbal (perpetration and victimization; 10 items) scale of the CADRI was administered. Frequency of each behavior in the last year was rated on a 4-point scale (“Never” to “Often (6 or more times)”). Item averages were computed for each partner for each subscale. Verbal aggression scores were operationalized for each person as the maximum of her/his reported perpetration and their partner’s reported victimization, thus taking both partners’ reports into account. The CADRI yields significant agreement between partners, good test-retest stability, and correlates with observed couple behavior [68].
3. Areas of conflict. The Conflict Assessment Tool-Kiddie (KCAT) was adapted from Capaldi [4] and previous research from our lab [69], with additional items added to capture issues in adolescent relationships. This questionnaire was designed to prompt the couple with personally relevant issues (e.g., trusting each other, what to do when we hang out, too much attention to other girls’ pictures and posts on social media), so that they felt prepared to discuss relationship problems later in the problem-solving task. For each item, participants were instructed to select how important it is that their partner make a change on a 6-point scale from “Not at all important” to “Very important” so that the most salient issues could be identified to discuss.
4. Observational coding. All observational tasks were coded with the Rapid Marital Interaction Coding System, 2^nd^ generation (RMICS-2) [70,71]. Across studies representing more than 1,000 couples, the RMICS has demonstrated discriminative, convergent, predictive, and construct validity [70]. In RMICS-2, behavior is coded in continuous 5-second intervals as belonging to one of 7 categories: High Intensity Hostility, Low Intensity Hostility, Constructive Problem Discussion, Low Intensity Positivity, High Intensity Positivity, Dysphoric Affect, Other. Only one of the 7 codes was assigned to each interval. If more than one code was present during a speaker turn, a theoretically derived hierarchy (that is, prioritizing negative codes, then positive codes, then neutral codes) determined which code to assign. Low-Intensity Hostility (for example, angry affect, criticism, and negative mind reading) summary scores were calculated for each person based on the percentage of his or her total intervals scored as “hostile (low-intensity).”

Trained coders were blind to study hypotheses and had no access to any information about the couples. Coders undergo an intensive training process and are required to demonstrate substantial agreement (that is, several consecutive agreement scores of κ > 0.60 with a trainer on test videos) before being allowed to code study data. During the study data coding phase, coders met weekly with the trainer to review mutually coded videos and recalibrate as necessary to prevent drift. The mutually coded videos (that is, 25 percent of all interactions) were selected at random, and coders were kept blind to which videos were mutually coded. Interrater agreement was measured for each coder against the trainer. Interrater agreement for an intricate coding system was good (κ = 0.54, G = .81) by established standards [34,35]. Kappa is attenuated by low base rates and multiple coding categories; G provides a less biased reliability estimate for complex behavioral coding.

### 2.4. Operationalization

Hostility escalation and de-escalation operationalizations. Each partner’s displayed affect time series was converted to a sequential variable indicating whether one became, remained, or ceased being hostile from one 5-sec interval to the next during the topic discussions. Specifically, hostility was dichotomized as either escalating (i.e., changing from non-hostile to hostile) or not escalating (i.e., remaining either hostile or non-hostile, or decreasing in hostility) at time interval *t*, compared with interval *t*+1. Likewise, hostility was dichotomized as either de-escalating (i.e., changing from hostile to non-hostile) or not de-escalating (i.e., remaining either hostile or non-hostile, or escalating in hostility). The extent to which one partner negatively reinforced the other partner by de-escalating in hostility (vs. not de-escalating), following the other partner’s escalations in hostility, was estimated. That is, when one partner became hostile, how often did the other partner negatively reinforce such behavior by backing down? This is shown as an antecedent-consequent table in Table 1, Panel A. We also examined the extent to which one partner’s hostile behavior was followed by similar hostile behavior from the other partner (Table 1, Panel B). Finally, the amount of hostile versus non-hostile behavior displayed during conflict was calculated as the ratio of the total amount of time that each partner displayed hostile versus non-hostile behavior during conflict discussions. The hostile-to-nonhostile ratio scores were then log transformed to retain the rank order among the scores while spreading out the scores for individuals who displayed less hostile to nonhostile behavior during conflict (*n* = 125, 29.9%) and pulling in the scores for individuals who displayed more hostile to nonhostile behavior (*n* = 195, 46.7%).

### 2.5. Analytic Method

All analyses were conducted using Mplus Version 8.4 software [72]. The aggression variables were winsorized [73] to reduce the influence of outliers. Regarding missing data, psychological and physical aggression were measured for all study participants, and the rate of missingness for observational data was only 1.91%, reflecting video file corruption for six couples’ interactions. However, 87.6% (*n* = 183) of couples had one or more conflicts during the problem-solving interaction tasks and thus had codable observational data on which to estimate coercive processes during couples’ interactions (i.e., negative reinforcement and reciprocity).

For negative reinforcement, sequential dependences in one partner’s hostility de-escalations (vs. not) following the other partner’s escalations (vs. not) were examined using multilevel log-linear models (MLM; [74,75]). For negative reciprocity, a MLM was examined in which sequential dependences in one partner’s hostility escalations (vs. not) following the other partner’s escalations (vs. not) were simultaneously estimated for each partner using data across the topic discussion interactions.

Displayed hostility escalations and de-escalations were incorporated in the MLM using effects coding for the antecedent-consequent tables given in Table 1 (Howe, Dagne & Brown, 2005). First, the aggregate frequencies of behaviors during the couples’ interaction in the antecedent-consequent tables (i.e., *a, b, c*, and *d* from Table 1) denoted time, level-1. Second, a row contrast (i.e., *a –b* from Table 1) denoted whether a partner was more likely to de-escalate versus not de-escalate in hostility following the other partner’s hostile escalation. Negative reinforcement parameters reflect the tendency for one partner to de-escalate versus not de-escalate hostility, given the other partner had escalated versus did not escalate in hostility. The row contrasts reflect each partner’s negative reinforcement of the other, denoting level-2 parameters (treated as random effects and free to correlate). This allowed for between-subjects variability in the tendency to be negatively reinforced for hostility escalations. For negative reciprocity, a similar MLM was estimated, denoting the tendency for one partner’s hostile behavior to be followed by similar hostile behavior from their partner (see Table 1, Panel B).

Hypotheses pertain to level-2 parameters from the negative reinforcement and reciprocity MLMs. To retain cases with missing data on the coercive process variables, a two-step approach was used in which each partner’s MLM factor scores of negative reinforcement and negative reciprocity were first estimated and saved, and then subsequently correlated with the aggression variables. The main hypotheses were addressed by testing whether the negative reinforcement and reciprocity factor scores were significantly associated with physical and psychological aggression. That is, we examined whether those who were more likely to be negatively reinforced by their partners’ de-escalations in hostility following their partner’s escalations were more likely to perpetrate psychological and physical aggression (H1). Likewise, we examined whether those who were more likely to engage in negative reciprocity with their partners were more likely to perpetrate psychological and physical aggression (H3). Further, we examined whether the overall ratio of hostile to non-hostile behavior exhibited during conflict was associated with perpetration of psychological and physical aggression (H2).

## 3. Results

**Figure 1.**
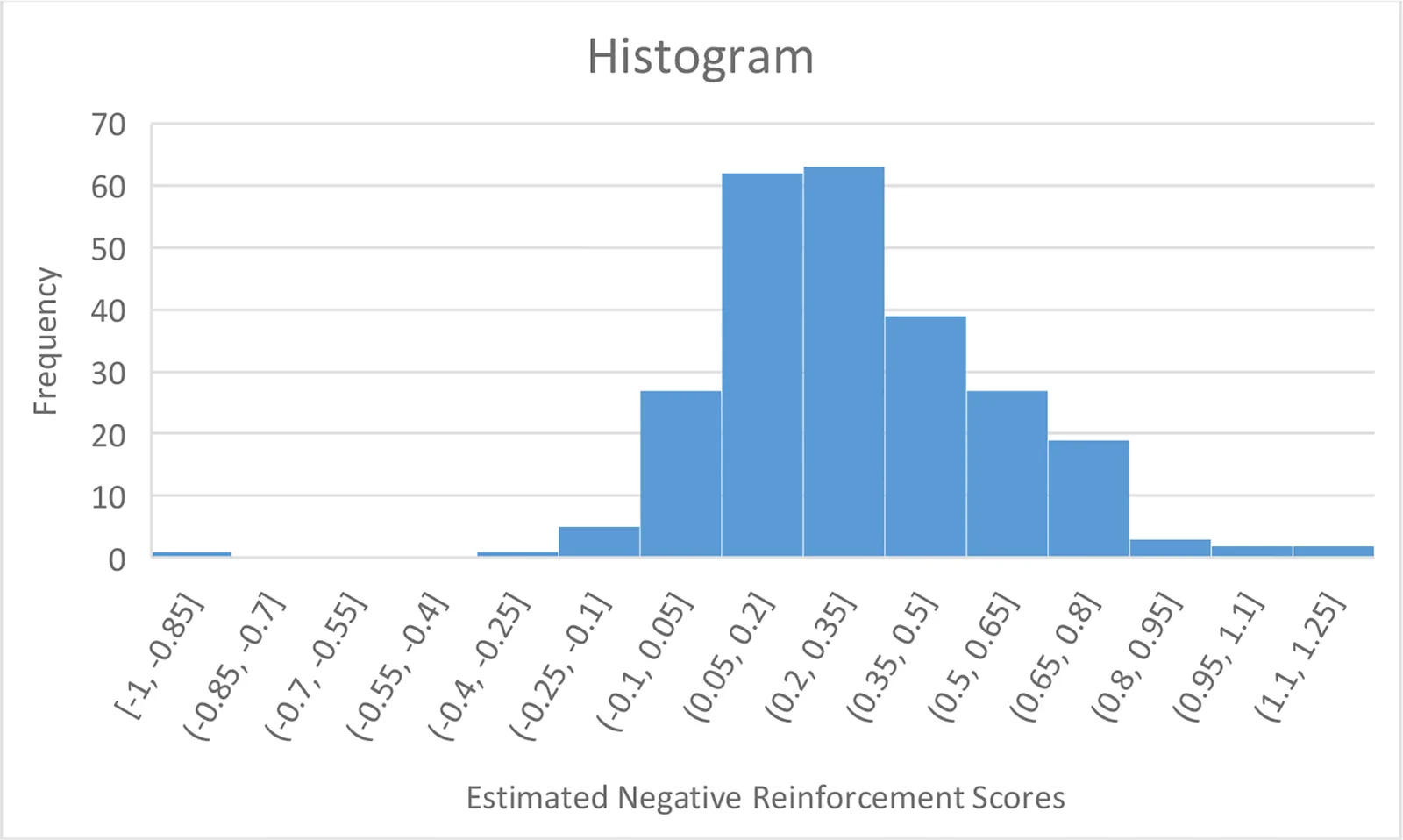

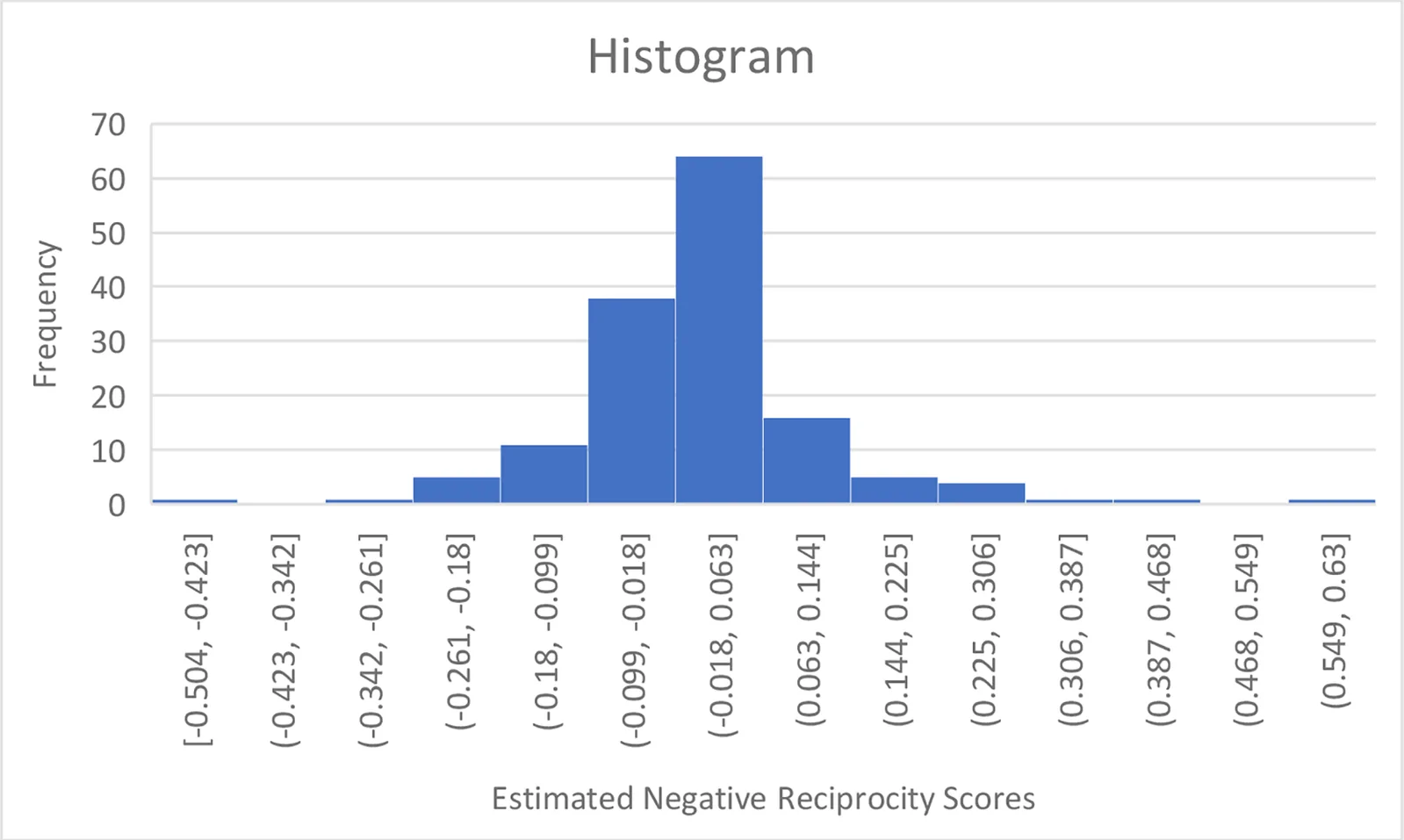
Histograms of the estimated negative reinforcement and negative reciprocity scores from the MLM.

The distributions of the estimated negative reinforcement and negative reciprocity scores from the MLM are depicted in Figure 1. Results indicated significant heterogeneity in negative reinforcement and negative reciprocity scores across the sample (unstandardized variance component (*SE*) =.064 (.008) for negative reinforcement and .007 (.002) for negative reciprocity). In addition, partners’ negative reinforcement scores were significantly positively associated (*r* = .299, *p* = .008), whereas their negative reciprocity scores were not (*r* = .158, *p* = .104). Finally, it should be noted that as a sensitivity check, we also examined an alternate operationalization for negative reinforcement such that the antecedent at time *t-1* was defined as escalating in hostility vs. *de-escalating* (compared to escalating in hostility vs *not de-escalating* [i.e., remained hostile or non-hostile; or de-escalated in hostility). The estimated negative reinforcement scores based on these two operationalizations were very highly correlated (*r* = .97, *p* < .001), thus further validating the scores.

Next, to test our hypotheses, we examined correlations among the coercion and aggression variables. Results are presented in Table 2. For both psychological and physical aggression, small positive significant associations were found between perpetration of aggression and the tendency for one partner to be negatively reinforced for hostility escalations by their partners’ de-escalations (vs. not) (H1), whereas no associations between aggression and negative reciprocity reached significance (H3). Perpetration of psychological and physical aggression were each significantly positively associated with the ratio of hostile vs non-hostile behavior during conflict. Thus, those who were more likely to be hostile vs. non-hostile during conflict were significantly more likely to perpetrate psychological and physical aggression. It is important to note that although H1 and H2 were supported, the estimated associations were quite small and none of the models accounted for a significant amount of explained variance in teen dating violence (for negative reinforcement: *R^2^(SE)* = 0.017 (0.013), *p* = 0.208 for psychological aggression and *R^2^(SE)* = 0.025 (0.016), *p* = 0.103 for physical aggression; for the ratio of hostile to non-hostile behavior: *R^2^(SE)* = 0.017 (0.012), *p* = 0.157 for psychological aggression and *R^2^(SE)* = 0.013 (0.009), *p* = 0.158 for physical aggression). Finally, all aggression variables were significantly positively related to each other, both within and across partners (see Table 3). This indicates that individuals who perpetrated more psychological aggression toward their partners were also likely to have perpetrated more physical aggression, and that those who perpetrated aggression were likely to have partners who also perpetrated aggression.

## 4. Discussion

This study represents a test of Patterson’s “basic training” coercion hypothesis in TDV. The findings indicate that negative reciprocity in and of itself did not predict the presence of TDV; proportions of hostile behavior and the negative reinforcement of hostility both significantly did for both psychological and physical TDV. This replicates previous findings regarding hostility [4] and highlights negative reinforcement as a possible mechanism in TDV.

Negative reciprocity is more descriptive than mechanistic. This tendency for an individual to respond in kind or escalate in response to their partner’s hostile overture was predictive of aggression and injuries in a longitudinal study by Capaldi and colleagues [4]. However, our finding that negative reciprocity was not associated with violence replicates one of the only other micro-coded observational studies of coercion in adolescent romantic relationships. Ha et al. (2019) found that the reciprocation of negative behavior by adolescent partners during conflict discussions was not predictive of TDV [76]. Rather, these researchers found that failure to reciprocate positivity during conflict (i.e., responding to a positive overture with hostility, negativity, or neutrality) mediated the relationship between conflict and later TDV in adolescent couples [76]. This further aligns with our finding regarding the importance of the proportion of hostile to nonhostile behavior displayed during conflict as predictive. Moreover, from a developmental perspective, the latter study sample (of couples between the ages of 13 and 18 [*M* = 16.5, *SD* = .99]) assessed at two time points 6 months apart is more akin to ours than Capaldi’s, which followed participants from late adolescence through their mid-twenties. Indeed, adolescents’ lack of experience navigating romantic conflict in the context of partnerships that are objectively fragile and normatively essential may render the inability to engage with a partner in positive problem-solving a greater risk factor for aggression [76,77]. Indeed, responding in kind to partner negativity during conflict may be within developmental norms across much of adolescence, only serving as a risk factor when it reflects the absence of expected interpersonal maturation during emerging adulthood.

The association between relative reinforcement of escalating hostility and TDV may be critical. It is a mechanistic process that is consistent with the matching law. The matching law provides a theoretical framework for understanding behavior, underlies all applied behavior analysis and its success as a treatment, and is at the heart of Patterson’s seminal work [41]. Notably, negative reinforcement patterns were correlated between partners in this sample, consistent with the notion that adolescent partners may mutually train one another to escalate conflict by rewarding escalating behavior with de-escalation.

In accordance with equifinality, a developmental cascade perspective suggests different pathways to TDV in romantic partnerships. For select children, TDV and associated hostile partner escalation represent yet a new developmental manifestation of the trajectory from early and severe conduct disorder to perpetration of bullying to deviant peer affiliations (in this case, facilitating assortative mating) and eventually to severe adult domestic violence [78]. For these adolescents, who tend to engage in delinquency, relationships are significantly more conflictual and intense than for typical adolescents [79]. Moreover, hostility, criticism, and aggression are characteristic, and these relationships facilitate increases in the severity of both partners’ deviant behavior [79]. Life-course persistent antisociality, however, is likely to be among the rarer explanations for TDV.

For most teens, the family context is among the most important contributors to adolescent romantic relationships. Findings that older adolescents who have higher quality healthy relationships with their parents [80,81] and whose parents have higher quality marital relationships [82], have higher quality relationships, are well replicated. Indeed, stage theories of romantic relationships have long suggested that when starting to date, adolescents test out and employ impersonal strategies learned in their relationships with peers and parents, as well as from observing their parents’ interactions [83]. For highly conflictual and coercive families, this can manifest in adolescents not learning to regulate anger, failing to learn effective interpersonal problem-solving skills (perhaps including learning to override the impulse to respond to negativity in kind), and relying on aggression [82,84]. Research provides support for this prospect. In one study, dysfunctional behaviors observed during couples’ marital conflict discussions were significantly associated with a host of dysfunctional behaviors observed during their adolescent children’s discussions with their own romantic partners. Specific dysfunctional behaviors included verbal aggression, negativity, withdrawal, low cohesion, and ineffective problem solving [85].

Further, longitudinal research found that observed hostility in parents’ marital relationships and reported hostility in close friendships predicted hostility three years later in older adolescents’ romantic partnerships [86]. The family environment seems to be such a crucial contextual support for interpersonal development that changes to it, even during adolescence, can be impactful. Indeed, in another study, adolescents’ hostile and aggressive behavior at baseline was associated with less effective interpersonal problem-solving skills [81]. However, greater degradation in the family climate during adolescence (a certain amount of which is typical during this stage) was associated with increased adolescent hostility (implicitly, a lack of socioemotional maturation), which in turn predicted their relationship violence [84].

Finally, sociocognitive and underlying neurobiological developmental factors may make adolescence a period of susceptibility for using dysfunctional interpersonal strategies (e.g., reinforced hostility) and aggression. Puberty ushers in a period of profound neurobiological restructuring, including reorganization of the brain areas underlying emotional responsivity, reward-sensitivity, and executive function (Blakemore & Mills, 2014). Additional peripubertal changes are evident in enhanced reactivity of the stress-responsive hypothalamic pituitary adrenal (HPA) axis [87], with social-interpersonal stress particularly salient [88]. As a result, adolescents seem to experience stress uniquely during this period [87], are more emotionally reactive and impulsive, and have less inhibitory control than during any other period across development [42,89,90]. Aligned with findings on social stress, rejection sensitivity hits its apex during adolescence [91]. These factors, in combination with the realities that (a) social competence is normatively tenuous in relation to social demands from early to mid-adolescence [92] and (b) adolescent romantic relationships are highly conflictual [93] put adolescents at increased risk for displaying patterns consistent with reinforced hostility. As a sensitive period for socioemotional maturation [42], however, adolescence also offers a window during which interpersonal and aggressive behaviors are particularly malleable [84], and thus, effective intervention holds promise.

This study has several notable strengths. It draws on a large and racially diverse sample of 209 adolescent dating couples, providing a rare and rich dataset from a generalizable sample. Second, the multi-method assessment contributes to a rigorous design that adds observational, turn-by-turn coding of escalation and de-escalation behaviors to self-report data, offering insights into dynamics that the participants themselves are unlikely to be aware of (and thus couldn’t report on). Further, sequential coding coupled with multilevel modeling offers a sophisticated, nuanced examination of rapidly unfolding, sometimes subtle, interpersonal processes (i.e., escalation and de-escalation dynamics).

The study has several important limitations. First, the cross-sectional design precludes a firm causal inference between patterns of negative reinforcement and the emergence, increase, or maintenance of aggressive behaviors. Second, reliance on laboratory tasks means that we may not have captured a purely naturalistic conflict, nor did we observe actual aggression. Some couples did not engage in observable conflict during laboratory tasks. Additionally, as noted earlier, our micro-temporal operationalization of negative reinforcement captures moment-to-moment yielding rather than conflict termination, a methodological adaptation of Patterson’s original model necessitated by the fact that many couples did not ever terminate their conflicts in this setup. The RMICS hierarchical coding rule (prioritizing hostile codes when multiple behaviors co-occur within an interval) reflects the established primacy of negativity in relationship distress (35) but necessarily collapses within-interval behavioral complexity. Additionally, our TDV measurement approach combines self-reported perpetration with partner-reported victimization to reduce under-reporting. Although this approach has advantages, it does not distinguish between perpetration and victimization roles within the dyad and thus does not allow inferences about directionality of violence. Finally, our sample was drawn from an urban and suburban population in the New York City metropolitan area, which may limit generalizability to rural or less diverse populations.

Our findings have implications for research and prevention. With respect to research, the clear next step is to extend these findings with designs that can speak to longitudinal course, and ultimately etiological importance. Future research should test whether negative reinforcement processes temporally precede TDV increases, as coercion theory would predict. Incorporating daily diary or ecological momentary assessment methods could complement the laboratory design and offer more naturalistic findings. Such methods would help establish whether the coercive patterns observed in our structured laboratory tasks generalize to spontaneous conflicts in natural environments.

If research does reveal that coercion is a causal mechanism contributing to TDV, then it will be important to place it within a larger cascade of influences. This would include understanding how reinforcement of hostile behavior in one’s family as a child affects one’s risk of becoming involved with a dating partner who has a similar (or different) reinforcement history. Identifying additional potential moderators (e.g., self-regulatory abilities, relationship qualities) could help us understand for whom and under what circumstances reinforcement processes are most impactful.

The implications of our findings for the prevention of TDV are particularly compelling. Negative reinforcement emerges from this study as a potentially useful prevention target. Current curricula addressing healthy dating behaviors in adolescents (e.g., *Safe Dates* [65], *Shifting Boundaries* [94]) do teach general conflict resolution skills and how to recognize escalation, but they do not teach how these escalation behaviors can be rewarded and why that may contribute to aggression. Programs could augment general skills with information on the coercive process, accessibly highlighting the trajectory from seemingly trivial negativity to escalating hostility, explaining reinforcement through conflict endings. Preventionists may consider including both the tendency to yield to hostile escalation and the inability to reciprocate positive engagement in conflict problem-solving as risk factors to be modified. Helping adolescents recognize and change nuanced maladaptive communication patterns could potentially modify those processes before they become ingrained. Ideally, prevention developers could teach adolescents micro-skills for mutually stepping down during conflict. Additionally, if research suggests coercive conflict in dating relationships is related to the same patterns with teens and parents, interventions aimed at altering coercive conflict at home might spill over and generalize to dating.

In conclusion, this study reveals negative reinforcement of hostile escalation, more than mere reciprocity of hostility, is potentially a dyadic mechanism linking conflict processes to teen dating violence (likely among other dyadic mechanisms). This study used turn-by-turn dynamics of escalation and de-escalation to examine coercion in relationships [54], as opposed to strictly conceptualizing coercion in relation to conflict termination. It appears that this more microscopic approach to conceptualizing interpersonal coercive process still reveals its significant links to aggression. Problematic coercive processes can occur in the momentary changes throughout a disagreement—not simply at its ending—offering opportunities to intervene with gentler step-down strategies. Moreover, these results add to an accumulating literature devoted to understanding the utility of coercive dynamics in close relationships. Unlike many theories of adult intimate partner violence (IPV), the coercive process likely operates across development. As a first foray into romantic partnerships during a period when socioemotional competence is normatively tenuous, adolescent romantic relationships are distinct from those of adults, and many theories of adult IPV (which do not have to account for peers, context, and profound neuro-biological and socioemotional development) have been found lacking [95].

## Figures and Tables

**Table 1.** Antecedent contingency tables for negative reinforcement and negative reciprocity.

*Panel A. Negative reinforcement during conflict*.		
	Partner	
Own	De-escalated in hostility at time t + 1	Did not de-escalate in hostility at time t + 1
Escalated in hostility at time t	*a*	*b*
Did not escalate in hostility at time t	*c*	*d*
*Panel B. Negative reciprocity during conflict*.		
	Partner	
Own	Hostile at time t + 1	Not hostile at time t + 1
Hostile at time t	*a*	*b*
Not hostile at time t	*c*	*d*

**Table 2.** Hypothesis test results: Estimated correlations between aggression and coercion variables.

	Psychological aggression	Physical aggression
Negative reinforcement during conflict (H1)	0.129 (0.054), p = 0.016	0.159 (0.055), p = 0.004
Negative reciprocity during conflict (H3)	−0.039 (0.050), p = 0.440	−0.009 (0.034), p = 0.797
Ratio hostile to non-hostile behavior during conflict (H2)	0.130 (0.047), p = 0.005	0.112 (0.040), p = 0.005

Note: Table values denoted r(SE), p.

**Table 3.** Correlations among perpetration of psychological and physical aggression.

Association	Estimated *r* (standard error), p-value
Own psychological and own physical aggression	0.502 (0.078), p < 0.001
Own and partner’s psychological aggression	0.729 (0.078), p < 0.001
Own and partner’s physical aggression	0.757 (0.136), p < 0.001
Own psychological and partner’s physical aggression	0.471 (0.078), p < 0.001

Note: Table values denoted r(SE), p.

## Data Availability

Datasets are available upon request from the corresponding author.

## Abbreviations

The following abbreviations are used in this manuscript:

TDV: Teen Dating Violence
CADRI: Conflict in Adolescent Dating Relationships Inventory
KCAT: Conflcit Assessment Tool – Kiddie
RMICS: Rapid Marital Interaction Coding System
MLM: Multilevel log-linear models
